# Genetic mapping and candidate gene analysis for melon resistance to *Phytophthora capsici*

**DOI:** 10.1038/s41598-020-77600-2

**Published:** 2020-11-24

**Authors:** Pingyong Wang, Xiaojun Xu, Guangwei Zhao, Yuhua He, Chong Hou, Weihu Kong, Jian Zhang, Shuimiao Liu, Yongyang Xu, Zhihong Xu

**Affiliations:** Zhengzhou Fruit Research Institute, Chinese Academy of Agricultural Sciences, Zhengzhou, 450009 Henan China

**Keywords:** Plant sciences, Genetics, Agricultural genetics, Plant breeding, Plant genetics

## Abstract

*Phytophthora* blight is one of the most serious diseases affecting melon (*Cucumis melo*) production. Due to the lack of highly resistant germplasms, the progress on disease-resistant research is slow. To understand the genetics of melon resistance to *Phytophthora capsici*, an F_2_ population containing 498 individuals was developed by crossing susceptible line E31 to highly resistant line ZQK9. Genetic analysis indicated that the resistance in ZQK9 was controlled by a dominant gene, tentatively named *MePhyto*. Through bulked-segregant analysis (BSA-Seq) and chromosome walking techniques, the *MePhyto* gene was mapped to a 52.44 kb interval on chromosome 12. In this region, there were eight genes and their expression patterns were validated by qRT-PCR. Among them, one wall-associated receptor kinase (WAK) gene *MELO3C002430* was significantly induced in ZQK9 after *P. capsici* inoculation, but not in E31. Based on the non-synonymous mutation site in *MELO3C002430*, a cleaved amplified polymorphic sequence (CAPS) marker, CAPS2430, was developed and this maker was co-segregated with *MePhyto* in both F_2_ population and a collection of 36 melon accessions. Thus *MELO3C002430* was considered as the candidate gene and CAPS2430 was a promising marker for marker-assisted selection (MAS) in breeding. These results lay a foundation for revealing the resistance mechanism of melon to *P. capsici*.

## Introduction

*Phytophthora* blight, caused by *Phytophthora capsici*, is one of the most serious soil-borne diseases affecting melon (*Cucumis melo*) and other cucurbits in the world. *P. capsici* infects plants at any growth stage, resulting in necrosis of the roots, stems, and leaves, as well as fruit rot, leading to substantial yield reductions^1–3^. *P. capsici* overwinters in the soil in the form of oospores, which are able to endure extreme environmental conditions, including desiccation and cold temperatures, and can survive in the soil for many years, even without a host plant^4,5^. High temperature and humidity condition is conducive to the spread of this disease, which poses a huge challenge to protected cultivation. Traditionally, chemical pesticide application is considered as the immediate control method. But the food safety risks caused by pesticide residues are getting more and more attention. Applying resistant cultivars is the most effective and environmentally-friendly approach^6^. So it is urgently needed to carry out the research on resistance genes and further apply them to disease-resistance breeding.

Research on plant resistance to this disease has been conducted as early as the middle of the nineteenth century. In potato, more than thirty major or qualitative *R* genes have been identified from diverse *Solanum* species. Some of these *R* genes have been cloned, and part of them were introgressed into cultivated strains^7,8^. In soybean, more than twenty resistance genes have been identified^9^, and research on mining new genes is still in progress. In tomato, both qualitative and quantitative resistance genes have been reported. Three major resistance genes, *Ph-1*, *Ph-2* and *Ph-3*, were identified in the wild species and successfully introgressed into the commercial cultivars^10,11^. For cucurbits, only a few reports on the resistance to *P. capsici* have been conducted, mainly due to the lack of germplasm resources with high resistance. Padley et al. identified a squash breeding line, # 394-1-27-12, with high resistance to *P. capsici* and predicted that its resistance was conferred by three dominant genes^12^. Donahoo et al. screened three melon lines, PI 420180, PI 176936, and PI 176940, with high resistance to *P. capsici*^6^. Pontes et al. found two melon accessions, CNPH-093 and L040, which exhibited high resistance to *P. capsici*^13^. In previous study, we identified one melon inbred line, ZQK9, which was highly resistant to *P. capsici*^14^. In this study, we investigated the inheritance mode of resistance trait in ZQK9 and firstly mapped the resistance gene. Candidate gene was predicted and marker designed based on the mutation site in candidate gene was validated to be promising in marker-assisted selection (MAS) for melon breeding.

## Results

### Inheritance of *P. capsici* resistance in ZQK9

**Figure 1:**
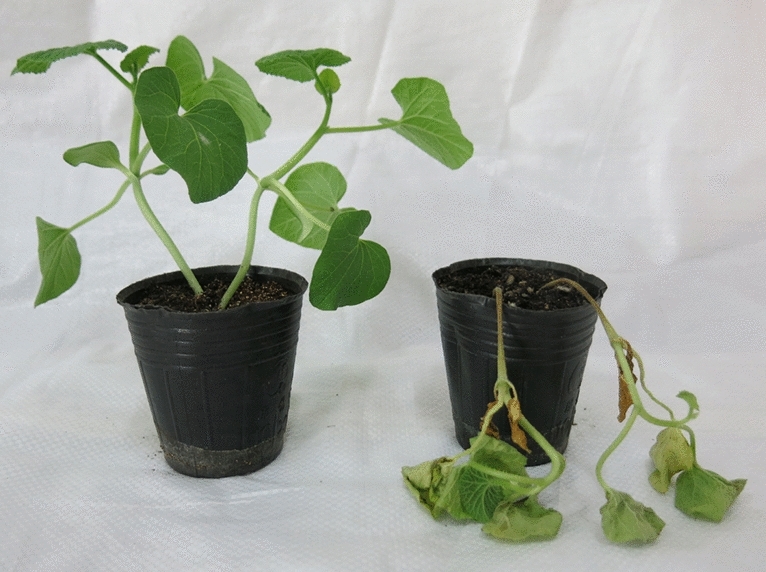
Phenotypes of ZQK9 (left) and E31 (right) 7 days post-inoculation.

The two parental lines, ZQK9 (male parent, P_2_) and E31 (female parent, P_1_), clearly displayed differential reactions to *P. capsici*. ZQK9 exhibited high resistance with no symptom after inoculation, while E31 exhibited water-soaked lesions with a slight brown color on the primary root at 3 days post-inoculation (dpi) and plants died within 1 week (Fig. 1). At 10 dpi, all individuals of the F_1_ and BC_1_P_2_ populations were highly resistant to *P. capsici*, indicating that the resistance in ZQK9 was completely dominant. The resistant and susceptible plants in the F_2_ population segregated as 374:124, fitting a 3:1 segregation ratio (χ^2^ = 0.0027 < 3.84, p = 0.05). The resistant and susceptible plants in the BC_1_P_1_ population segregated as 60:43, following a 1:1 segregation ratio (χ^2^ = 2.49 < 3.84, p = 0.05). These results suggested that a single dominant gene, tentatively designated as *MePhyto*, conferred resistance to *P. capsici* in ZQK9.

### Gene location association analysis by BSA-Seq

**Figure 2:**
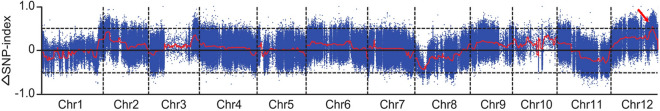
The result of gene location association analysis using BSA-seq. The red arrow indicated the initial mapping region.

Two parental and two F_2_ genomic DNA pools, including the RP (ZQK9), SP (E31), F_2_R (resistant), and F_2_S (susceptible) pools, were constructed for the bulked-segregant analysis sequencing (BSA-Seq) using Illumina high-throughput sequencing technique. A total of 39,723,071, 44,025,572, 78,697,860, and 54,916,327 clean reads were obtained from the RP, SP, F_2_R, and F_2_S pools, respectively. Roughly 80% of the clean reads were mapped to the melon genome, resulting in ~ 80% coverage with at least 10 × depth in the four pools (Supplementary Table S1). There were 2,510,000 different single-nucleotide polymorphisms (SNPs) between the RP and SP pools. Each identified SNP was used to compute the SNP-index. The ΔSNP-index graph was plotted and computed against the genome positions by combining information from the F_2_R and F_2_S pools’ SNP-indexes (Fig. 2). SNP analysis indicated that the region encompassing the genomic positions 22,075,687–25,394,539 on chromosome 12 may be the candidate location of *MePhyto*. In this region, 5,455 InDel sites were screened and 2,027 pairs of InDel primers were designed according to the following parameters: 55 °C ≦ Tm ≦ 60 °C, 15 bp ≦ primer length ≦ 21 bp, 100 bp ≦ product size ≦ 280 bp.

### Mapping of the *MePhyto* gene

**Figure 3:**
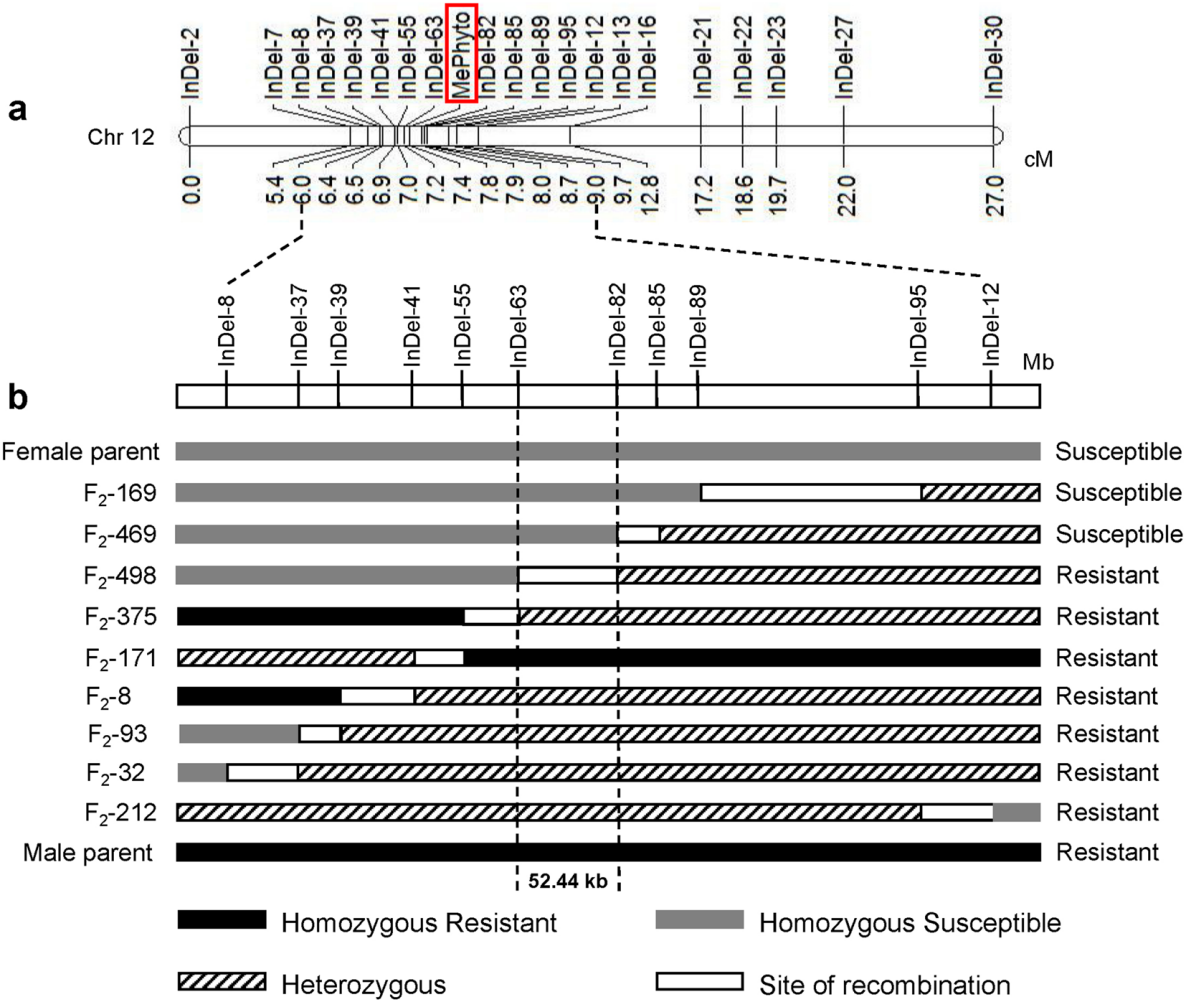
The linkage and partial physical maps of melon resistance gene *MePhyto* on chromosome 12. (**a**) Genetic linkage map constructed according to F_2_ population with 20 markers. (**b**) Partial physical map of the genomic region between InDel-8 and InDel-12 and the graphical genotype of the recombinants in F_2_. *MePhyto* was narrowed to a 52.44 Kb region between InDel-63 and InDel-82 by analyzing the genotypes and phenotypes of nine recombinants.

To precisely map the locus, the designed InDel primers were used to map the *MePhyto* gene. Markers were evaluated in the two parental lines to find clear polymorphisms, and then polymorphic markers were used to genotype the 498 individuals in the F_2_ population. Finally, a total of 98 InDel markers were evaluated and 20 markers were identified as potentially associated with resistance in ZQK9. Through linkage analysis, the *MePhyto* gene was mapped into a 0.6 cM genetic region between InDel-63 and InDel-82 markers, corresponding to the 22,861,058–22,913,498 bp region on chromosome 12 (Fig. 3). The BC_1_P_1_ and BC_1_P_2_ populations were used to verify the accuracy of mapping results by F_2_ population wtih the 11 InDel markers in Fig. 3b. It turned out that the mapping results of the backcross populations were consistent with that of the F_2_ population (Supplementary Table S2, S3).

According to the melon reference genome information^15^, the order of markers on the linkage map was consistent with their physical positions on chromosome 12 (Table 1). There were eight genes in this 52.44 kb genomic interval (Table 2). Among them, *MELO3C002430* was annotated as a wall-associated receptor kinase (WAK) gene, which was generally recognised to contribute to plant disease resistance.

**Table 1:** Primers of markers used in gene mapping and marker efficiency analysis.

Marker name	Position on chromosome 12	Primers (5′–3′)	
InDel-2	22091434–22091706	F: ACCATGCTTTACGGGTCGT	R: TGCACTAATCTCAGCTGCCC
InDel-7	22632575–22632844	F: GGAGGGCCATCCACCATC	R: AGTGCGGGGTTTCATTTCA
InDel-8	22663519–22663789	F: TGAGACGTTGCAAAGATG	R: TGCCTCTTGTGGGTTGCA
InDel-37	22706727–22706973	F: CCACGACCGTGCTTGGAA	R: ACGGTGGCACTTTCTCCG
InDel-39	22738688–22738962	F: CGCTCACGTGGTTCATTCT	R: AGGGGTGAAAGTGACAAGT
InDel-41	22764992–22765265	F: GGCGAATTGGAGCAGTGC	R: CCACTGGAGCAGAGGATCG
InDel-55	22821415–22821637	F: ACCAAAACCCAGTTACCGG	R: TGGAGTGGACAGGTCGGT
InDel-63	22861058–22861297	F: GCCGCTAGTTCACCAGCA	R: AGTCTAACAGAGAGCACGA
CAPS2430	22901899–22902619	F: TATGTAACTGCTATCTCCCT	R: GGTTACTGGAGCTTTGGCTC
InDel-82	22913276–22913498	F: TCAAGGGATGTCCAGTGGA	R: CTCAAACATTGGCCTGAGA
InDel-85	22922328–22922475	F: TGCTCGTGATGGATCTAGGA	R: CCTTCCAACCCACTCCACA
InDel-89	22939478–22939753	F: TCCGCTGGTGGATCTGGA	R: GGCTGATGCGTACGTCGT
InDel-95	23070558–23070708	F: GTTGTCGGTGCCTTCTACCA	R: CCCTCACTCTGTTGCCCC
InDel-12	23112364–23112563	F: TGGGGGATCTTGAGAAGCT	R: GGAAGAGCCTGCCTTGCA
InDel-13	23209843–23210093	F: GCAGAACTCGGCCTGGTT	R: CATCAACACTTTGGGGATG
InDel-16	23509247–23509466	F: GCTACTTTGTACAATTGGGC	R: TTGCATCGAGATGGCCCG
InDel-21	24004974–24005253	F: TGCTCCGCTGTAGCTTTTGT	R: ACCCCTTGATCAACGCTTGT
InDel-22	24103365–24103599	F: TCTTTCACGCCCCCACAC	R: ACACACGTAAACCAACGCA
InDel-23	24199343–24199588	F: GAGGTCGGTGCTTCGTCC	R: TCACCGGCGTTTACTGGC
InDel-27	24493802–24494080	F: TGCCGATGCATTCCAAGTTG	R: CAACGTTGCCCAATGCCA
InDel-30	24792064–24792315	F: TTGCCTTCACCATCATCATC	R: TACCTGGAGTACCCGCGG

**Table 2:** Genes located in the mapping region.

Gene ID	Physical location (5′ to 3′)	Functional annotation
*MELO3C002429*	chr12: 22,910,381–22,912,823	Ankyrin repeat family protein
*MELO3C002430*	chr12: 22902248–22903843	Wall-associated receptor kinase 3-like protein
*MELO3C002431*	chr12: 22900233–22901918	Pectate lyase
*MELO3C002432*	chr12: 22895401–22896784	Ankyrin repeat-containing protein
*MELO3C002433*	chr12: 22884326–22884968	uncharacterized protein
*MELO3C002434*	chr12: 22879435–22882064	Ankyrin repeat family protein
*MELO3C002435*	chr12: 22871856–22873860	Retrovirus-related Pol polyprotein from transposon TNT 1–94
*MELO3C002436*	chr12: 22867110–22868670	Enoyl-[acyl-carrier-protein] reductase

### Expression analysis of genes in target region

**Figure 4:**
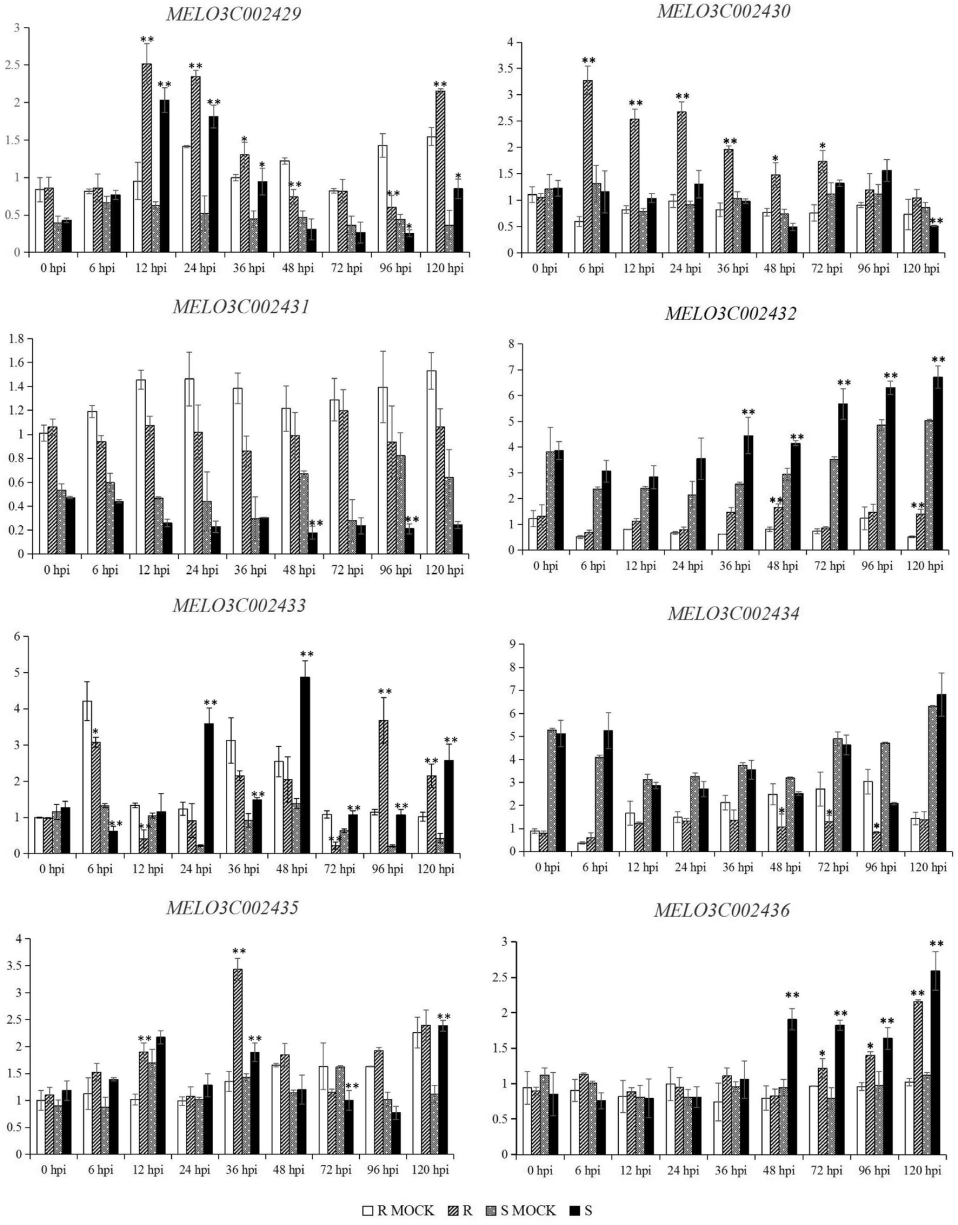
The expression pattern of genes in the mapping region at different time using qRT-PCR. R MOCK: ZQK9 inoculated with sterilized water. R: ZQK9 inoculated with zoospore suspension. S MOCK: E31 inoculated with sterilized water*.* S: E31 inoculated with zoospore suspension. Gene expression are normalized to actin. Data are displayed as the mean ± SD of three biological replicates. Significance levels are indicated as *p < 0.05 and **p < 0.01.

The expression pattern of the eight genes were conducted to analyze whether their expression levels were associated with disease resistance (Fig. 4, Supplementary Table S4). Compared to the corresponding control at each time point, *MELO3C002429*, *MELO3C002431* and *MELO3C002432* showed the similar expression pattern between ZQK9 and E31. *MELO3C002433* was continuously down-regulated from 6 to 72 h post-inoculation (hpi) and then up-regulated at 96 and 120 hpi in ZQK9, while in E31, it was down-regulated at 6 hpi, but continuously up-regulated from 12 to 120 hpi. *MELO3C002434* was not significantly induced in E31, but significantly (P < 0.05) down-regulated at 48, 72 and 96 hpi in ZQK9. *MELO3C002435* showed up-regulated expression pattern from 6 to 48 hpi in both ZQK9 and E31, but only significantly (P < 0.01) induced at 12 and 36 hpi in ZQK9 and 36 hpi in E31. In ZQK9, *MELO3C002435* was down-regulated at 72 hpi and up-regulated at 96 and 120 hpi, while in E31, *MELO3C002435* was down-regulated at 72 and 96 hpi but up-regulated at 120 hpi. In ZQK9, *MELO3C002436* was not significantly induced in the first 48 h, but significantly up-regulated from 72 to 120 hpi (P < 0.05). In E31, *MELO3C002436* was significantly induced to be up-regulated from 48 to 120 hpi (P < 0.01). *MELO3C002430* showed the continuously up-regulated expression pattern in ZQK9, but presented no significantly different expression level until 120 hpi (down-regulated) in E31. In ZQK9, *MELO3C002430* was triggered quickly by *P. capsici* inoculation, reaching a peak expression level soon at 6 hpi, and maintained a high expression level in the first 72 h. This specifically up-regulated expression pattern of *MELO3C002430* in ZQK9 indicated it may be related to the disease resistance.

### Sequence variance analysis of *MELO3C002430* and marker efficiency verification

**Figure 5:**
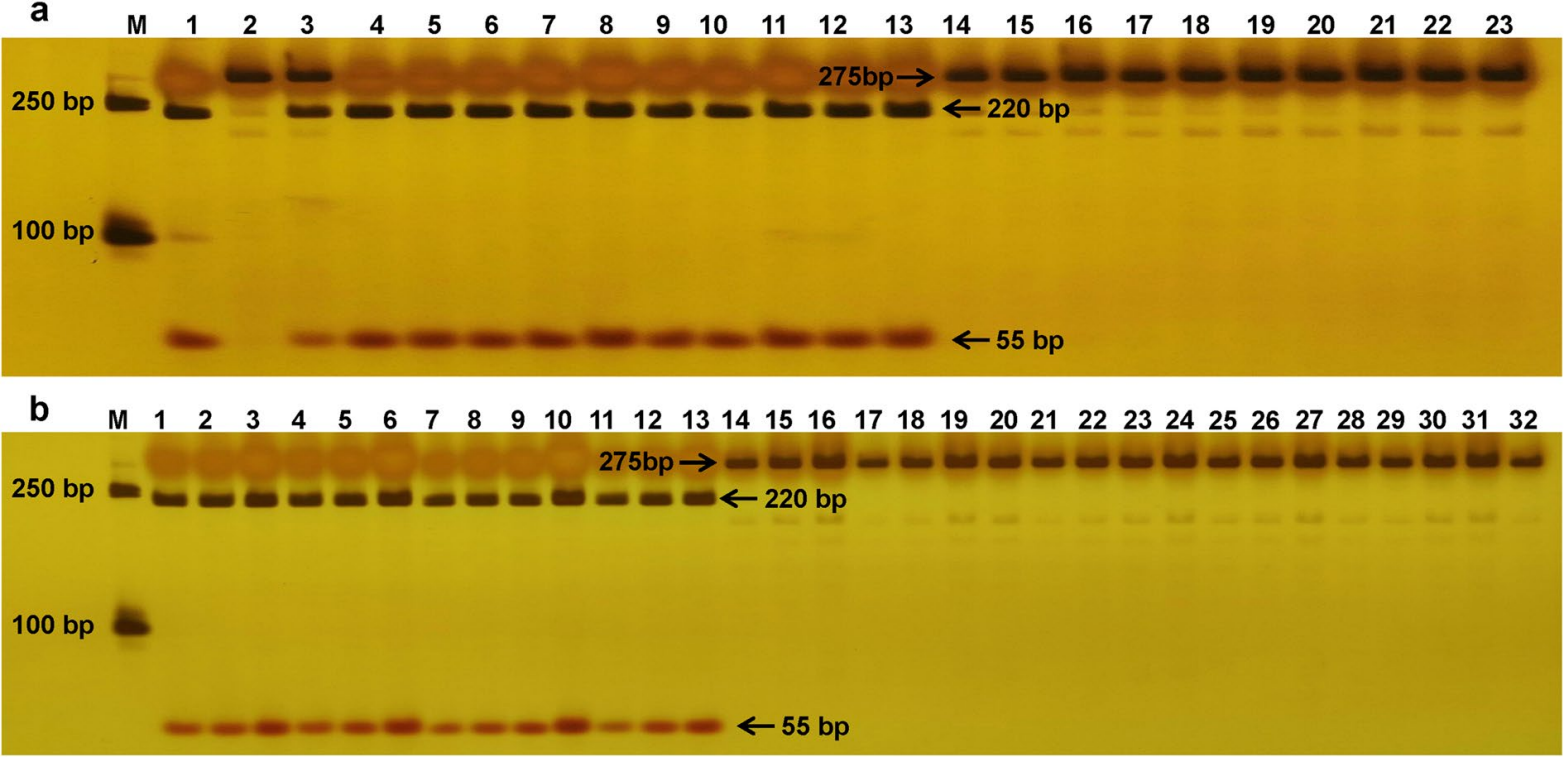
Digested PCR fragments of maker CAPS2430 by restriction enzyme *Fau I*. (**a**) M: DL2000 DNA Marker, Lane 1: ZQK9, Lane 2: E31, Lane 3: F_1_ (E31 × ZQK9), Lane 4–13: 10 resistant F_2_ individuals, Lane 14–23: 10 susceptible F_2_ individuals. (**b**) M: DL2000 DNA Marker, Lane 1–13: 13 resistant melon accessions, Lane 14–32: 19 susceptible melon accessions.

To verify the difference of *MELO3C002430* between ZQK9 and E31, the CDS sequence of *MELO3C002430* was cloned using designed specific primers 2430-CDS-F/R (Supplementary Table S4). The sequence alignment indicated that there were five nucleotide substitutions, and only one non-synonymous substitution at position + 890 resulted in changes in the 297th amino acid residues, from glycine (GGT) in ZQK9 to alanine (GCT) in E31. Using dCAPS Finder v2.0 software (http://helix.wustl.edu/dcaps/dcaps.html), one cleaved amplified polymorphism makers (CAPS) named CAPS2430 was designed based on the non-synonymous SNP site in *MELO3C002430*. Its amplified products in ZQK9 can be digested into two bands (220 bp and 55 bp) by the restriction enzyme *Fau I*, while in E31, the amplified products can not be digested (Fig. 5a). The F_2_ population and a panel of 36 accessions were employed to verify the co-segregation between CAPS2430 and the resistance trait. As a result, CAPS2430 was fully co-segregated in all individuals of the F_2_ population and can accurately identify the 36 melon accessions (Fig. 5b; Tables 3, 4), suggesting that the SNP site in *MELO3C002430* was conserved among different melon accessions, and *MELO3C002430* was a strong candidate gene of *MePhyto*.

**Table 3:** Genotype of 498 F_2_ individuals identified by CAPS2430 marker.

Genotype	Phenotype	Number of F_2_ individuals
Homozygous dominant	Resistant	150
Heterozygous	Resistant	224
Homozygous recessive	Susceptible	124

**Table 4:** Melon accessions used for marker efficiency analysis.

Accessions	Origin	Disease index (%)		Phenotype^a^	Genotype
		1st	2nd		CAPS2430^b^
ZQK9	Henan, China	0	0	R	G:G
PI 140766	Iran	1	0	R	G:G
PI 143217	Iran	1	2	R	G:G
PI 164409	India	1	2	R	G:G
PI 381760	India	1	3	R	G:G
PI 164395	India	2	3	R	G:G
Cuimi	Henan, China	3	1	R	G:G
PI 140627	Iran	3	1	R	G:G
B66	Henan,China	3	6	R	G:G
PI 614328	India	4	7	R	G:C
B121	Henan, China	5	3	R	G:G
B31	Henan, China	5	6	R	G:G
B26	Henan, China	7	6	R	G:G
PI 140774	Iran	9	7	R	G:G
PI 137854	Iran	9	10	R	G:G
Shilenghuangjingua	Zhejiang, China	22	30	R	G:C
PI 165525	India	32	38	R	G:C
Fenghuangxuan-2	Henan, China	45	40	R	G:C
PI 164466	India	63	70	S	C:C
PI 140678	Iran	74	69	S	C:C
PI 614581	India	76	71	S	C:C
Jiangsusanyegua	Jiangsu, China	77	79	S	C:C
Yadanqing	Jiangsu, China	77	83	S	C:C
Qingpitianrou	Jiangsu, China	80	85	S	C:C
Sanyehuasugua	Jiangsu, China	80	85	S	C:C
Zhenjianghuangjingua	Jiangsu, China	82	77	S	C:C
PI 182938	India	87	92	S	C:C
PI 614417	India	88	90	S	C:C
PI 143244	Iran	89	90	S	C:C
Jingnong 4	Hubei, China	90	92	S	C:C
PI 136228	India	91	92	S	C:C
Daxianggua	Zhejiang, China	92	89	S	C:C
Hongzimatisu	Jiangsu, China	95	93	S	C:C
Hongpicui	Jiangsu, China	95	97	S	C:C
PI 165515	India	100	100	S	C:C
Huangxianggua	Hunan, China	100	100	S	C:C
PI 614395	India	100	100	S	C:C
E31	Henan, China	100	100	S	C:C

1st, the first resistance evaluation experiment. 2nd, the second resistance evaluation experiment.

^a^Phenotype designation: (R) resistant phenotype, (S) susceptible phenotype.

^b^Genotype designation of CAPS2430: (G:G) homozygous disease-resistant genotype, (G:C) heterozygous genotype, (C:C) homozygous susceptible genotype.

### Protein structure analysis of MELO3C002430

**Figure 6:**
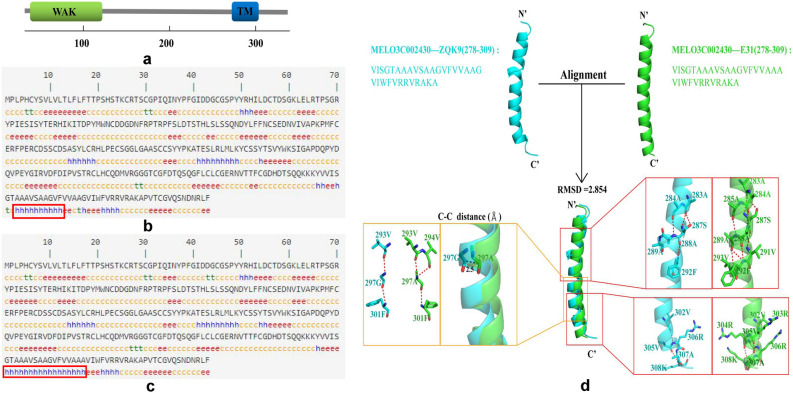
Protein structure analysis of MELO3C002430. (**a**) Domain region of the protein. WAK, wall-associated receptor kinase; TM, transmembrane region. (**b**, **c**) Secondary structure of the protein in ZQK9 and E31, respectively. h, alpha helix; e, extended strand; t, beta turn; c, random coil. Red boxes indicate the alpha helixes with different number in MELO3C002430 protein of ZQK9 and E31. (**d**) The difference of the protein tertiary structure in the mutation region generated by PyMOL molecular graphic system version 2.4 (https://pymol.org/2/). Red dotted line represents the hydrogen bond.

In order to understand the difference of MELO3C002430 protein in ZQK9 and E31, the conserved domain was analyzed using the the NCBI conserved domain search tool and SMART software. One wall-associated receptor kinase domain was detected between the amino acids 26–127, and one transmembrane region was located between the amino acids 280–302 (Fig. 6a). The protein secondary structure was predicted using SOPMA software. It was found that the mutation of 297th amino acid resulted in a different number of alpha helices between the amino acids (aa) 278–297 (Fig. 6b,c).

In order to further compare and analyze the structural differences in the mutation region, the tertiary structure of 32 residues in the amino acid positions 278–309 was predicted using the SWISS-MODEL server. The residues in ZQK9, namely MELO3C002430(278–309)-ZQK9, was homologous with human epidermal growth factor structure model (PDB code: 2N5S) with the sequence similarity of 29%, while the residues in E31, namely MELO3C002430(278–309)-E31, was homologous with T-cell surface glycoprotein CD3 delta chain model (PDB code: 6JXR) with the sequence similarity of 25%.

Both models were mainly composed of alpha helices, and the orientation of amino acid residues was relatively consistent. The residues MELO3C002430(278–309)-ZQK9 have two fewer helices than MELO3C002430(278–309)-E31, which were located at 287S-289A and 306R-308 K. By analyzing the hydrogen bonds of the two segments, we found the residue 297G in ZQK9 formed hydrogen bonds with 293 V and 301F, while 297A in E31 formed hydrogen bonds with 293 V, 294 V, and 301F (Fig. 6d).

## Discussion

*P. capsici* is a destructive pathogen which has caused widespread and devastating damage to the melon industry^4^. In our previous study, one melon inbred line, ZQK9, was identified as highly resistant to *P. capsici*^14^. Using ZQK9 and another highly susceptible line E31 as the paternal and maternal lines, we constructed the F_1_, F_2_, and BC_1_ populations and found the resistance in ZQK9 was controlled by a dominant gene, tentatively named *MePhyto*.

Through the combination of BSA-Seq and chromosome walking techniques, we successfully mapped the *MePhyto* gene into a 52.44 kb interval on chromosome 12. One WAK gene *MELO3C002430* was triggered quickly by *P. capsici* inoculation, reaching a peak expression level soon at 6 hpi, and maintained a high expression level in the first 72 h. The CAPS marker CAPS2430 designed based on non-synonymous SNP site in *MELO3C002430* was co-segregated with the resistance trait in the F_2_ mapping population and other 36 melon accessions. Therefore, it can be presumed that *MELO3C002430* is likely a strong candidate gene underlying *MePhyto.* Marker CAPS2430 was a promising molecular marker for marker-assisted selection in melon breeding.

WAK is a type of receptor protein kinase located in the cell wall that sense extracellular and intracellular signals. WAKs have a receptor-like protein structure and contain extracellular, transmembrane, and intracellular domains. The extracellular domain is constructed from several epidermal growth factor (EGF) repeats and binds to the transmembrane domain, forming a bond with the cell wall^16^. The intracellular domain of serine-threonine kinase plays a role in activating signaling cascades^17^. WAKs bind pectin polymers or pectin fragments that are released by mechanical damage or pathogen infection. This in turn triggers signaling, thereby activating host basal resistance^18,19^. In rice, the *OsWAK1* gene was significantly induced during incompatible interactions with *Magnaporthe oryzae*. Overexpression of *OsWAK1* enhanced the plant resistance^20^. In tomato, the WAK gene *SlWAK1* was identified as one of the flagellin-induced inhibition by effector (FIRE) genes. Plant receptor recognition of microbial-associated molecular patterns (MAMP) triggered the expression of *SlWAK1*, thereby activating a sustained immune response^21^. After *P. capsici* inoculation, *MELO3C002430* was triggered quickly reaching a peak expression level soon. As a receptor kinase gene, it might play an important role in sensing pathogen infection and activating downstream disease-resistant responses.

*P. capsici* is a kind of oomycetes. After inoculation, zoospores attach to the surface of host cells and invade into the host cells, then propagate in large quantities, which eventually lead to the disintegration of host cells^14^. Yu et al. observed the infection process of *P. capsici* on pepper leaves by scanning electronic microscopy and found that the zoospores attached to the surface of leaves and differentiated rapidly to form adhesive cysts within 3 h. Germ tubes were germinated from the cysts at about 12 hpi, and started to penetrated the pepper leaf at about 24 hpi. Within the first 24 h, there was no obvious difference between the resistant and susceptible lines. The obvious differences appeared at 3 dpi as the hypha perforated the leaves of susceptible line and propagated in large quantities, which was not observed in resistant line^22^. In this study, no visible disease symptom appeared on susceptible E31 until 3 dpi. This suggested that the infection process in melon and pepper is similar. The continuously up-regulation pattern within 72 hpi in ZQK9 during *P. capsici* infection process further confirmed the possibility of *MELO3C002430* as a candidate resistance gene.

The protein structure analysis revealed that the amino acid mutation in *MELO3C002430* was located in the transmembrane region between the amino acids 280–302, which caused a quantitative change in the alpha helix of the protein secondary structure. A previous study confirmed that specific amino acid mutations in the WAK gene changed the ability of the gene to gather with downstream acting factors^23^. The prediction results of the protein tertiary structure indicated that 297G in MELO3C002430 from ZQK9 could form hydrogen bonds with 293 V and 301F, while 297A in MELO3C002430 from E31 could form hydrogen bonds with 293 V, 294 V, and 301F. There were two fewer helical structures in ZQK9 than in E31, which were located in 287S-289A and 306R-308 K, respectively. Based on the hydrogen bonding analysis, we found that the lack of helical structures may be related to the number of hydrogen bonds and the inconsistency of residues. These structural differences may affect the combination of *MELO3C002430* and downstream genes. These results will be helpful in elucidating the mechanism of melon resistance to *P. capsici* and in marker-assisted selection for practical breeding.

## Materials and methods

### Plant materials and genetic mapping population

The resistant melon line ZQK9 was obtained from the National Mid-term GenBank for Watermelon and Melon, Zhengzhou Fruit Research Institute, Chinese Academy of Agricultural Sciences (ZFRI, CAAS). The line exhibited high resistance to *P. capsici* with no symptom after inoculation. An inbred melon line E31 developed by ZFRI was highly susceptible to *P. capsici*. To understand the genetic basis of resistance to *P. capsici* in ZQK9 and map resistance genes, an F_2_ population containing 498 individuals was developed by crossing E31 (female parent, P_1_) to ZQK9 (male parent, P_2_). Three additional populations, F_1_, BC_1_P_1_ (F_1_ × E31), and BC_1_P_2_ (F_1_ × ZQK9), were also developed to explore the genetic model of disease resistance in ZQK9. These populations respectively contained 72, 103 and 105 individuals. Ten plants of two parent lines were used for pathogen inoculation and genomic DNA pools construction.

Seeds of the two parents, F_1_, F_2_, BC_1_P_1_, and BC_1_P_2_ were sown in plastic bowls (9 × 7 × 8 cm) filled with a mixture of steam-sterilized peat and vermiculite (1:1). Plants were grown in an artificial climate chamber under a 16/8 h light/dark photoperiod and 28 °C/20 °C day/night air temperature. Seedlings with two fully expanded true leaves were used for *P. capsici* inoculation.

### Inoculum preparation and inoculation

*P. capsici* was isolated from infected melon plants collected from Hainan province, China, and verified through morphological identification and PCR-based detection as described before^24^. Purified hypha of *P. capsici* was transferred to PDA medium in petri dishes (9 cm diameter) and incubated at 25 °C in the dark for 5 d. A piece of medium with hypha (1 cm diameter) from the edge of the colony was transferred to carrot agar medium for spore production at 25 °C in the dark for 5 d, and then under 12/12 h light/dark for 5–7 d. Mycelia in each petri dish was soaked with sterilized water and incubated at 4 °C for 40–60 min. Then, petri dishes were placed under light for 40 min at 25 °C for zoospore release. The zoospore suspension was removed by a pipette and filtered through two layers of gauze. The concentration of the zoospore suspension was determined by a hemacytometer and diluted to 1 × 10^6^ mL^-1^ prior to inoculation. Before inoculation, seedlings were watered until the soil was saturated. Then, 1 mL of zoospore suspension was inoculated on the soil surface around each seedling’s primary root.

### Disease evaluation

Ten days after inoculation, the disease resistance level of each plant was determined according to the modified criteria based on previous report^25^. Levels ranged as 0–5: 0 = no visible symptom; 1 = water-stained and slightly dehydrated lesions at the base of stem, and plants did not collapse or wilt; 2 = lesions progressed upwards, but did not exceed the cotyledon section, causing constriction at the stem base, and cotyledons and true leaves did not wilt; 3 = lesions progressed upwards and exceeded the cotyledon section, plants collapsed with apparent wilted cotyledons, and true leaves did not wilt; 4 = long and brownish lesions were present on the stems, lesions were extended and dehydrated, all leaves except the uppermost leaves were defoliated, and plants almost died; 5 = plants died. Plants scored as 0 or 1 were classified as resistant, while those scored as 2–5 were classified as susceptible.

### DNA extraction and genome pools construction

Young leaves from the two parental lines and experimental populations were collected for DNA extraction. Genomic DNA was extracted using a modified CTAB method^26^. DNA concentrations were determined using a NanDrop-1000 spectrophotometer (Thermo Fisher, Willmington, DE, USA). Genomic DNA pools of the two parental and two F_2_ pools with extreme phenotypes were constructed for the BSA-Seq analysis, including the RP (ZQK9), SP (E31), F_2_R (resistant), and F_2_S (susceptible) pools. The two parental pools were constructed by mixing an equal concentration of DNA from 10 ZQK9 and 10 E31 plants, respectively. The F_2_R and F_2_S pools were constructed by mixing an equal concentration of DNA from 22 resistant (score = 0) and 14 susceptible (score = 5) F_2_ individuals, respectively.

### Whole genome resequencing data analysis

Quantified DNA samples were randomly fragmented into 350 bp lengths by a Covaris ultrasonic breaker (Covaris, East Sussex, UK) for library construction. Illumina libraries for the two parental and two F_2_ pools were prepared according to the manufacturer’s instructions, including fragmentation, adapter ligation, size selection, and PCR enrichment. Paired-end sequencing of the fragments was performed using an Illumina NovaSeq sequencing platform (Illumina, San Diego, CA, USA). Short reads from the four libraries were aligned to the reference melon (DHL92) genome (Version 3.5.1, http://cucurbitgenomics.org/) using BWA software^27^. Sequence alignment file conversions were performed using SAM tools^28^. SNP and InDel mining were performed using GATK software^29^. Additionally, the localization (e.g., upstream, downstream, or intergenic regions) and coding effects (e.g., synonymous or non-synonymous mutations) of SNPs were annotated using SnpEff software^30^. Before the association analysis, SNPs were selected according to the following criteria:

1. SNPs were homozygous and incongruous in the parental lines;
2. Depth of the SNPs in the two parental pools was > 5 × ;
3. SNP-index ≧ 0.3 and SNP depth > 7 in the two F_2_ pools;
4. SNPs whose SNP-index was not missing in the two F_2_ pools were selected.

A collection of high-quality SNP markers were obtained for the association analysis.

### Gene location association analysis

The SNP-index and ΔSNP-index were calculated to detect significant differences in the frequency of genotypes between DNA pools. The SNP-index was calculated as the proportion of reads harboring the SNP in the F_2_R or F_2_S pools relative to the two parental pools. Then, the ΔSNP-index was defined by subtracting the F_2_R pool SNP-index from the F_2_S pool SNP-index.

The ΔSNP-index = 0 if the SNP index of the F_2_S pool was equal to the F_2_R pool. If the ΔSNP index = 1, one genotype was associated almost entirely with the F_2_S pool and the associated SNPs were therefore linked closely to the susceptible phenotype. In contrast, if the ΔSNP index = –1, the associated SNPs were linked to the resistant phenotype. Candidate region was identified when the ΔSNP-index of markers were above the threshold (ΔSNP-index = 0.5) at the 95% of confidence interval^28,31^. The ΔSNP-index plot regression curve was obtained using R scripts (https://www.r-project.org).

### Molecular marker development for linkage map construction

Polymorphic SNP and InDel sites in the candidate region were used to develop molecular markers. Primers for each polymorphic site were designed using Primer Premier v5.0 (Premier Biosoft Ltd., Palo Alto, CA, USA). dCAPS Finder v2.0 software (http://helix.wustl.edu/dcaps/dcaps.html) was used to convert SNP markers to CAPS or derived cleaved amplified polymorphism (dCAP) markers^32,33^, which were detected with the appropriate restriction enzyme purchased from New England Biolabs (Beverly, MA, USA). Alternatively, if no endonuclease was suitable for the SNP site, then PCR reaction products were amplified and sequenced to directly identify SNPs. Markers were initially evaluated in the parental lines to uncover clear polymorphisms and polymorphic markers (Table 1) were used to genotype individuals in the F_2_ population. Amplification products of all markers were separated on 7% polyacrylamide gel. The genetic linkage map was constructed using JoinMap v4.0 software with a logarithm of odds (LOD) threshold = 3.0^34^.

### Expression analysis of genes in target region

Gene expression patterns in the mapping region were analyzed by qRT-PCR. Appropriate primers (Supplementary Table S4) for each gene were designed for quantitative analysis using Primer Premier v5.0 software. ZQK9 and E31 seedlings were inoculated with zoospore suspension. The control plants were inoculated with the same volume of sterilized water. Root samples were collected from the ZQK9 and E31 lines at 0, 6, 12, 24, 48, 72, 96, and 120 hpi. Three biological replicates were collected at each time point and for each replicate, root samples from three individual plants were mixed. Total RNA was extracted using an RNA simple Total RNA Kit (DP441, Tiangen, Beijing, China). Reverse transcription of RNA to cDNA was conducted using a PrimeScript RT reagent Kit with a gDNA Eraser (RR047A, TaKaRa, Beijing, China). Primer specificity was evaluated by BLAST searches against the melon genome and via the melt curve analysis through qRT-PCR amplification. PCR amplifications were performed on a Roche LightCycler 480 RT-PCR System (Roche Diagnostics, Rotkreuz, Switzerland) using a SYBR Premix Ex Taq Kit (RR420A, Takara, Beijing, China). Expression levels of the selected genes were normalized to the melon actin gene, *MELO3C023264*, and analyzed using the 2^-ΔΔCt^ method^35^.

### Sequence variance analysis and candidate gene prediction

The CDS of candidate genes were cloned in ZQK9 and E31. Primers used for homology-based cloning were designed according to the CDS database of melon DHL92 (http://cucurbitgenomics.org/organism/) (Supplementary Table S4). The genes were amplified by PCR using 2 × Phanta Max Master Mix (Vazyme, Nanjing, China); then, the amplified products were sequenced (Shanghai Sangon Biotech Co., Ltd) and aligned by DNAMAN software version 6 (http://www.biologydir.com/dnaman-info-1940.html). According to SNPs of genes, the CAPS or dCAPS markers were designed using dCAPS Finder 2.0 (http://helix.wustl.edu/dcaps/dcaps.html) and Primer Premier 5.0 (http://www.premierbiosoft.com/primerdesign/).

All of the 498 F_2_ individual plants were used to analyze the linkage relationship of CAPS marker and resistance traits. If linkage was observed, the markers were then tested in 36 melon accessions introduced from the Mid-term GenBank in ZFRI. Responses of the 36 accessions to *P. capsici* were evaluated using the method described above. Twenty plants of each line were planted and inoculated. Disease index (DI) was calculated as: DI = [∑ (number of diseased plants in the index × di)/(total number of plants investigated × highest di)] × 100%. A line was considered susceptible when DI > 50 and resistant when DI ≦ 50^36^. The experiment was independently repeated twice.

### Protein structure analysis of MELO3C002430

The gene conserved domain analysis was performed using NCBI conserved domain search tool (https://www.ncbi.nlm.nih.gov/structure). The exact location of different domains was predicted using SMART software (http://smart.embl-heidelberg.de/). The protein secondary structures were predicted using SOPMA method (CNRS, Lyon, France). The protein tertiary structures were predicted and by amino acid homology modeling using the SWISS-MODEL server (http://swissmodel.expasy.org/repository/) based on the existing structures in Protein Data Bank (PBD, https://www.rcsb.org/). DNAMAN software version 6 (http://www.biologydir.com/dnaman-info-1940.html) was used to analyze the differences among these molecules and PyMOL molecular graphic system version 2.4 (https://pymol.org/2/) was used for figure preparation.

## Supplementary information

Supplementary Information 1.

## Data Availability

The sequencing data of this study has been deposited in the NCBI Sequence Read Archive under the accession number PRJNA648669.
