# Supplementary material for: Genetic mapping and candidate gene analysis for melon resistance to Phytophthora capsici
Source: Sci Rep. 2020 Nov 24;10:20456. doi: 10.1038/s41598-020-77600-2 (PMC7686303; doi:10.1038/s41598-020-77600-2)
Supplement: Supplementary file 1 — Supplementary Information 1. [file 41598_2020_77600_MOESM1_ESM.pdf]

Genetic mapping and candidate gene analysis for melon resistance to *Phytophthora capsici*

Pingyong Wang†[1], Xiaojun Xu†[1], Guangwei Zhao[1], Yuhua He[1], Chong Hou[1], Weihu Kong[1], Jian Zhang[1], Shuimiao Liu[1], Yongyang Xu*[1], Zhihong Xu*[1]

† Pingyong Wang and Xiaojun Xu contributed equally to this work.

1 Zhengzhou Fruit Research Institute, Chinese Academy of Agricultural Sciences, Zhengzhou, Henan 450009, China

Author information: Pingyong Wang, E-mail: wangpingyong@caas.cn； Xiaojun Xu, E-mail: xuxiaojun@caas.cn; Guangwei Zhao, E-mail: zhaoguangwei@caas.cn; Yuhua He, E-mail: heyuhua@caas.cn; Chong Hou, E-mail: houchong9756@163.com; Weihu Kong, E-mail: kongweihu@caas.cn; Jian Zhang, E-mail: zhangjian03@caas.cn; Shuimiao Liu, E-mail: liushuimiao1234@163.com; Yongyang Xu, E-mail: xuyongyang@caas.cn; Zhihong Xu, xuzhihong@caas.cn.

First authors:

Pingyong Wang, E-mail: wangpingyong@caas.cn;

Xiaojun Xu, E-mail: xuxiaojun@caas.cn.

*** Corresponding authors:**

Zhihong Xu, E-mail: xuzhihong@caas.cn, Tel: +86-0371-65330930;

Yongyang Xu, E-mail: xuyongyang@caas.cn, Tel: +86-0371-65330930.

Supplementary Table S1. Summary of the sequencing data.

Library	Number of raw reads	Number of clean reads	Mapped reads	Mapping rate	Depth
RP	55,307,940	39,723,071	31,999,546	0.81	11.7
SP	65,731,966	44,025,572	34,949,086	0.8	12.79
F_2R	118,435,402	78,697,860	63,030,174	0.8	23.06
F_2S	79,746,448	54,916,327	40,620,473	0.74	14.86

Supplementary Table S2 Genotype analysis of the 11 markers located in *MePhyto* flanking region by BC_1P_1 population.

Code of BC_1P_1 indivaduals	Genotype of markers											
	InDel-8	InDel-37	InDel-39	InDel-41	InDel-55	InDel-63	*MePhyto*	InDel-82	InDel-85	InDel-89	InDel-95	InDel-12
1	a	a	a	a	a	a	a	a	a	a	a	a
2	h	h	h	h	h	h	c	h	h	h	h	h
3	h	h	h	h	h	h	c	h	h	h	h	h
4	a	a	a	a	a	a	a	a	a	a	a	a
5	h	h	h	h	h	h	c	h	h	h	h	h
6	h	h	h	h	h	h	c	h	h	h	h	h
7	a	a	a	a	a	a	a	a	a	a	a	a
8	a	a	a	a	a	a	a	a	a	a	a	a
9	a	a	a	a	a	a	a	a	a	a	a	a
10	a	a	a	a	a	a	a	a	a	a	a	a
11	h	h	h	h	h	h	c	h	h	h	h	h
12	h	h	h	h	h	h	c	h	h	h	h	h
13	a	a	a	a	a	a	a	a	a	a	a	a
14	h	h	h	h	h	h	c	h	h	h	h	h
15	a	a	a	a	a	a	a	a	a	a	a	a
16	h	h	h	h	h	h	c	h	h	h	h	h
17	a	a	a	a	a	a	a	a	a	a	a	a
18	h	h	h	h	h	h	c	h	h	h	h	h
19	a	a	a	a	a	a	a	a	a	a	a	a
20	h	h	h	h	h	h	c	h	h	h	h	h
21	a	a	a	a	a	a	a	a	a	a	a	a
22	h	h	h	h	h	h	c	h	h	h	h	h
23	a	a	a	a	a	a	a	a	a	a	a	a
24	a	a	a	a	a	a	a	a	a	a	a	a
25	h	h	h	h	h	h	c	h	h	h	h	h
26	h	h	h	h	h	h	c	h	h	h	h	h
27	h	h	h	h	h	h	c	h	h	h	h	h
28	a	a	a	a	a	a	a	a	a	a	a	a
29	a	a	a	a	a	a	a	a	a	a	a	a

30	h	h	h	h	h	h	c	h	h	h	h	h
31	a	a	a	a	a	a	a	a	a	a	a	a
32	h	h	h	h	h	h	c	h	h	h	h	h
33	h	h	h	h	h	h	c	h	h	h	h	h
34	a	a	a	a	a	a	a	a	a	a	a	a
35	a	a	a	a	a	a	a	a	a	a	a	a
36	h	h	h	h	h	h	c	h	h	h	h	h
37	h	h	h	h	h	h	c	h	h	h	h	h
38	h	h	h	h	h	h	c	h	h	h	h	h
39	h	h	h	h	h	h	c	h	h	h	h	h
40	a	a	a	a	a	a	a	a	a	a	a	a
41	a	a	a	a	a	a	a	a	a	a	a	a
42	h	h	h	h	h	h	c	h	h	h	h	h
43	h	h	h	h	h	h	c	h	h	h	h	h
44	a	a	a	a	a	a	a	a	a	a	a	a
45	a	a	a	a	a	a	a	a	a	a	a	a
46	a	a	a	a	a	a	a	a	a	a	a	a
47	h	h	h	h	h	h	c	h	h	h	h	h
48	a	a	a	a	a	a	a	a	a	a	a	a
49	h	h	h	h	h	h	c	h	h	h	h	h
50	a	a	a	a	a	a	a	a	a	a	a	h
51	a	a	a	a	a	a	a	a	a	a	a	a
52	h	h	h	h	h	h	c	h	h	h	h	h
53	h	h	h	h	h	h	c	h	h	h	h	h
54	a	a	a	a	a	a	a	a	a	a	a	a
55	h	h	h	h	h	h	c	h	h	h	h	h
56	h	h	h	h	h	h	c	h	h	h	h	h
57	h	h	h	h	h	h	c	h	h	h	h	h
58	h	h	h	h	h	h	c	h	h	h	h	h
59	h	h	h	h	h	h	c	h	h	h	h	h
60	a	a	a	a	a	a	a	a	a	a	a	a
61	a	a	a	a	a	a	a	a	a	a	a	a
62	h	h	h	h	h	h	c	h	h	h	h	h
63	h	h	h	h	h	h	c	h	h	h	h	h

64	a	a	a	a	a	a	a	a	a	a	a	a
65	h	h	h	h	h	h	c	h	h	h	h	h
66	a	a	a	a	a	a	a	a	a	a	a	a
67	h	h	h	h	h	h	c	h	h	h	h	h
68	h	h	h	h	h	h	c	h	h	h	h	h
69	h	h	h	h	h	h	c	h	h	h	h	h
70	h	h	h	h	h	h	c	h	h	h	h	h
71	h	h	h	h	h	h	c	h	h	h	h	h
72	h	h	h	h	a	a	a	a	a	a	a	a
73	h	h	h	h	h	h	c	h	h	h	h	h
74	h	h	h	h	h	h	c	h	h	h	h	h
75	a	a	a	a	a	a	a	a	a	a	a	a
76	h	h	h	h	h	h	c	h	h	h	h	h
77	h	h	h	h	h	h	c	h	h	h	h	h
78	a	a	a	a	a	a	a	a	a	a	a	a
79	a	a	a	a	a	a	a	a	a	a	a	a
80	h	h	h	h	h	h	c	h	h	h	h	h
81	h	h	h	h	h	h	c	h	h	h	h	h
82	a	a	a	h	h	h	c	h	h	h	h	h
83	h	h	h	h	h	h	c	h	h	h	h	h
84	h	h	h	h	h	h	c	h	h	h	h	h
85	a	a	a	a	a	a	a	a	a	a	a	a
86	h	h	h	h	h	h	c	h	h	h	h	h
87	h	h	h	h	h	h	c	h	h	h	h	h
88	a	a	a	a	a	a	a	a	a	a	a	a
89	a	a	a	a	a	a	a	a	a	a	a	a
90	h	h	h	h	h	h	c	h	h	h	h	h
91	h	h	h	h	h	h	c	h	h	h	h	h
92	h	h	h	h	h	h	c	h	h	h	h	h
93	h	h	h	h	h	h	c	h	h	h	h	h
94	h	h	h	h	h	h	c	h	h	h	h	h
95	a	a	a	a	a	a	a	a	a	a	a	a
96	h	h	h	h	h	h	c	h	h	h	h	h
97	a	a	a	a	a	a	a	a	a	a	a	a

98	a	a	a	a	a	a	a	a	a	a	a	a
99	a	a	a	a	a	a	a	a	a	a	a	a
100	h	h	h	h	h	h	c	h	h	h	h	h
101	h	h	h	h	h	h	c	h	h	h	h	h
102	a	a	a	a	a	a	a	a	a	a	a	a
103	h	h	h	h	h	h	c	h	h	h	h	h

a, the homozygous genotye of markers and susceptible phenotype of indivaduals; h, the heterozygous genotype of markers; c, the resistant phenotype of indivaduals.

Supplementary Table S3 Genotype analysis of the 11 markers located in *MePhyto* flanking region by BC_1P_2 population.

Code of BC_1P_2 indivaduals	Genotype of markers											
	**InDel-8**	**InDel-37**	**InDel-39**	**InDel-41**	**InDel-55**	**InDel-63**	***MePhyto***	**InDel-82**	**InDel-85**	**InDel-89**	**InDel-95**	**InDel-12**
1	h	h	h	h	h	h	c	h	h	h	h	h
2	h	h	h	h	h	h	c	h	h	h	h	h
3	b	b	b	b	b	b	c	b	b	b	b	b
4	b	b	b	b	b	b	c	b	b	b	b	b
5	b	b	b	b	b	b	c	b	b	b	b	b
6	b	b	h	h	h	h	c	h	h	h	h	h
7	b	b	b	b	b	b	c	b	b	b	b	b
8	h	h	h	h	h	h	c	h	h	h	h	h
9	h	h	h	h	h	h	c	h	h	h	h	h
10	b	b	b	b	b	b	c	b	b	b	b	b
11	b	b	b	b	b	b	c	b	b	b	b	b
12	b	b	b	b	b	b	c	b	b	b	b	b
13	h	h	h	h	b	b	c	b	b	b	b	b
14	b	b	b	b	b	b	c	b	b	b	b	b
15	h	h	h	h	h	h	c	h	h	h	h	h
16	b	b	b	b	b	b	c	b	b	b	b	b
17	h	h	h	h	h	h	c	h	h	h	h	h
18	b	b	b	b	b	b	c	b	b	b	b	b
19	b	b	b	b	b	b	c	b	b	b	b	b
20	h	h	h	h	h	h	c	h	h	h	h	h
21	h	h	h	h	h	h	c	h	h	h	h	h
22	h	h	h	h	h	h	c	h	h	h	h	h
23	h	h	h	h	h	h	c	h	h	h	h	h
24	b	b	b	b	b	b	c	b	b	b	b	b
25	h	h	h	h	h	h	c	h	h	h	h	h
26	b	b	b	b	b	b	c	b	b	b	b	b
27	b	b	b	b	b	b	c	b	b	b	b	b

28	h	h	h	h	h	h	c	h	h	h	h	h
29	h	h	h	h	h	h	c	h	h	h	h	h
30	b	b	b	b	b	b	c	b	b	b	b	b
31	b	b	b	b	b	b	c	b	b	b	b	b
32	b	b	b	b	b	b	c	b	b	b	b	b
33	b	b	b	b	b	b	c	b	b	b	b	b
34	h	h	h	h	h	h	c	h	h	h	h	h
35	h	h	h	h	h	h	c	h	h	h	h	h
36	h	h	h	h	h	h	c	h	h	h	h	h
37	h	h	h	h	h	h	c	h	h	h	h	h
38	b	b	b	b	b	b	c	b	b	b	b	b
39	h	h	h	h	h	h	c	h	h	h	h	h
40	b	b	b	b	b	b	c	b	b	b	b	b
41	h	h	h	h	h	h	c	h	h	h	h	h
42	h	h	h	h	h	h	c	h	h	h	h	h
43	h	h	h	h	h	h	c	h	h	h	h	h
44	b	b	b	b	b	b	c	b	b	b	b	b
45	h	h	h	h	h	h	c	h	h	h	h	h
46	b	b	b	b	b	b	c	b	b	b	b	b
47	h	h	h	h	h	h	c	h	h	h	h	h
48	h	h	h	h	h	h	c	h	h	h	h	h
49	b	b	b	b	b	b	c	b	b	b	b	b
50	h	h	h	h	h	h	c	h	h	h	h	h
51	b	b	b	b	b	b	c	b	b	b	b	b
52	b	b	b	b	b	b	c	b	b	b	b	b
53	b	b	b	b	b	b	c	b	b	b	b	b
54	b	b	b	b	b	b	c	b	b	b	b	b
55	h	h	h	h	h	h	c	h	h	h	h	h
56	b	b	b	b	b	b	c	b	b	b	b	b
57	b	b	b	b	b	b	c	b	b	b	b	b

58	h	h	h	h	h	b	c	b	b	b	b	b
59	b	b	b	b	b	b	c	b	b	b	b	b
60	b	b	b	b	b	b	c	b	b	b	b	b
61	b	b	b	b	b	b	c	b	b	b	b	b
62	h	h	h	h	h	h	c	h	h	h	h	h
63	b	b	b	b	b	b	c	b	b	b	b	b
64	h	h	h	h	h	h	c	h	h	h	h	h
65	b	b	b	b	b	b	c	b	b	b	b	b
66	b	b	h	h	h	h	c	h	h	h	h	h
67	b	b	b	b	b	b	c	b	b	b	b	b
68	h	h	h	h	h	h	c	h	h	h	h	h
69	b	b	b	b	b	b	c	b	b	b	b	b
70	h	h	h	h	h	h	c	h	h	h	h	h
71	b	b	b	b	b	b	c	b	b	b	b	b
72	b	b	b	b	b	b	c	b	b	b	b	b
73	b	b	b	b	b	b	c	b	b	b	b	b
74	b	b	b	b	b	b	c	b	b	b	b	b
75	b	b	b	b	b	b	c	b	b	b	b	b
76	b	b	b	b	b	b	c	b	b	b	b	b
77	b	b	b	b	b	b	c	b	b	b	b	b
78	h	h	h	h	h	h	c	h	h	h	h	h
79	h	h	h	h	h	h	c	h	h	h	h	h
80	h	h	h	h	h	h	c	h	h	h	h	h
81	b	b	b	b	b	b	c	b	b	b	b	b
82	b	b	b	b	b	b	c	b	b	b	b	b
83	b	b	b	b	b	b	c	b	b	b	b	b
84	h	h	h	h	h	h	c	h	h	h	h	h
85	b	b	b	b	b	b	c	b	b	b	b	b
86	h	h	h	h	h	h	c	h	h	h	h	h
87	b	b	b	b	b	b	c	b	b	b	b	b

88	b	b	b	b	b	b	c	b	b	b	b	b
89	h	h	h	h	h	h	c	h	h	h	h	h
90	h	h	h	h	h	h	c	h	h	h	h	h
91	h	h	h	h	h	h	c	h	h	h	h	h
92	b	b	b	b	b	b	c	b	b	b	b	b
93	b	b	b	b	b	b	c	b	b	b	b	b
94	h	h	h	h	h	h	c	h	h	h	h	h
95	b	b	b	b	b	b	c	b	b	b	b	b
96	b	b	b	b	b	b	c	b	b	b	b	b
97	b	b	b	b	b	b	c	b	b	b	b	b
98	h	h	h	h	h	h	c	h	h	h	h	h
99	h	h	h	h	h	h	c	h	h	h	b	b
100	h	h	h	h	h	h	c	h	h	h	h	h
101	b	b	b	b	b	b	c	b	b	b	b	b
102	b	b	b	b	b	b	c	b	b	b	b	b
103	b	b	b	b	b	b	c	b	b	b	b	b
104	b	b	b	b	b	b	c	b	b	b	b	b
105	b	b	b	b	b	b	c	b	b	b	b	b

b, the homozygous genotye of markers and resistant phenotype of indivaduals; h, the heterozygous genotype of markers; c, the resistant phenotype of indivaduals.

Supplementary Table S4. Primers used in qRT-PCR and CDS cloning experiments.

Gene	Primers (5' to 3')
MELO3C002429	2429-QPCR-F: GGAGAACCTTCCAGAACCCG
	2429-QPCR-R: GGAGATGCCAACATTCCAACTAA
MELO3C002430	2430-QPCR-F: GCTCCTAAGCCAATGTTCTGC
	2430-QPCR-R: GCCCCAATGCTCTTCCAATA
	2430-CDS-F: ATGCCACTCCCTCATTGCTACT
	2430-CDS-R: TCAAAAAAGCCTATTGTCATTGC
MELO3C002431	2431-QPCR-F: ATGGACTCAAAATCCTCCCACCT
	2431-QPCR-R: TTAGCAAGGTTTGCCGATGAAA
MELO3C002432	2432-QPCR-F: CAAACCTTACTGGAGGGGAGA
	2432-QPCR-R: GAACGCCGCCGCAAAA
MELO3C002433	2433-QPCR-F: AGCCTTTTCAAGCAGCAAT
	2433-QPCR-R: CACTTCCCTTTACAGCATACCT
MELO3C002434	2434-QPCR-F: CAATACGAGTTACGGTGGTTTA
	2434-QPCR-R: CATGCACGAGTTTGCTGTG
MELO3C002435	2435-QPCR-F: CGTAAGGATGGTTCGGGTTT
	2435-QPCR-R: TTTTGTTCATTTGTATCGTCGTG
MELO3C002436	2436-QPCR-F: CCTCCGAACACGAGCATAGA
	2436-QPCR-R: TGAGTGAACGCAGACATAAACG
MELO3C023264 (Actin)	Actin-F: CCTGGTATCGCTGACCGTAT
	Actin-R: TACTGAGCGATGCAAGGATG
